# Impact of primary tumour location on efficacy of bevacizumab plus chemotherapy in metastatic colorectal cancer

**DOI:** 10.1038/s41416-018-0304-6

**Published:** 2018-11-29

**Authors:** Fotios Loupakis, Herbert I. Hurwitz, Leonard Saltz, Dirk Arnold, Axel Grothey, Quynh Lan Nguyen, Stuart Osborne, Jonathan Talbot, Stefanie Srock, Heinz-Josef Lenz

**Affiliations:** 1 Istituto Oncologico Veneto (IRCCS), Via Gattamelata, 64, 35128 Padova, PD Italy; 2 Duke University Medical Center, 10 Duke Medicine Circle, Durham, NC 27710 USA; 3 Memorial Sloan Kettering Cancer Center, 1275 York Avenue, New York, NY 10021 USA; 4 Asklepios Tumorzentrum Hamburg, AK Altona, Paul-Ehrlich-Str. 1, 22763 Hamburg, Germany; 5 Mayo Clinic, 200 1st St SW, Rochester, MN 55902 USA; 6 F. Hoffmann-La Roche, Ltd., Grenzacherstrasse 124, CH-4070 Basel, Switzerland; 7 University of Southern California, Norris Comprehensive Cancer Center, Keck School of Medicine, 1441 Eastlake Ave, Los Angeles, CA 90033 USA; 8 Present Address: Genentech, Inc., 1 DNA Way, South San Francisco, CA 94080 USA

**Keywords:** Oncology, Scientific community, Diseases, Cancer

## Abstract

**Background:**

Two first-line (1L) bevacizumab trials showed the prognostic value of primary tumour location in metastatic colorectal cancer (mCRC). In this retrospective subgroup analysis, further analysis of the predictive effect of bevacizumab is presented.

**Methods:**

Patients with sidedness information from two randomised phase III studies of bevacizumab + chemotherapy (CT) vs CT as 1L mCRC treatment were analysed retrospectively.

**Results:**

Sidedness was determined in 1590 (27% right and 73% left) of 2214 patients. Progression-free survival was improved with bevacizumab + CT vs CT in right-sided (HR = 0.75; 95% CI 0.61, 0.93; *p* = 0.008) and left-sided (HR = 0.76; 95% CI 0.67, 0.86; *p* < 0.001) mCRC (pooled analysis). Similarly, overall survival was numerically improved with bevacizumab + CT vs CT in right-sided mCRC (HR = 0.82; 95% CI 0.65, 1.03; *p* = 0.085), and significantly improved in left-sided mCRC (HR = 0.85; 95% CI 0.74, 0.98; *p* = 0.028).

**Conclusions:**

This analysis indicates that the effect of bevacizumab is independent of tumour location in mCRC.

## Background

Metastatic colorectal cancer (mCRC) is a heterogeneous disease increasingly characterised by chromosomal and molecular variations.^1^ CRCs arising from the right or left side of the colon have markedly different clinical characteristics, incidence rates, and gene expression profiles.^2–5^ Right-sided tumours are more likely to be characterised by *BRAF* mutations and high microsatellite instability, whereas left-sided tumours are commonly associated with chromosomal instability and overexpression of epidermal growth factor receptor ligands.^3^ Right- and left-sided tumours also differ with regard to their microbiomes and host-related factors. Mucosal microbiota organisation has been shown to be a distinct feature of right-sided tumours,^6^ and right-sided CRC is associated with older age and fewer surgeries for urgent indications.^7^

Recent reports have focused on differences in prognosis and therapeutic outcome between tumours originating in the right and left side of the colon.^1,8,9^ In a report of two randomised phase III mCRC trials with bevacizumab, overall survival (OS) was significantly longer in left-sided vs right-sided tumours (AVF2017g: hazard ratio (HR) = 0.55, 95% confidence interval (CI) = 0.43–0.70; NO16966: HR = 0.71, 95% CI = 0.62–0.82); these differences remained statistically significant within treatment subgroups (i.e., bevacizumab + chemotherapy (CT) or CT alone).^8^

Given the fundamental differences in colon tumour sidedness, further understanding of the influence of sidedness from pivotal mCRC studies may add value to our current knowledge of optimal mCRC treatments. Here we present data on the benefit of adding bevacizumab to CT in patients with right- or left-sided tumours from the NO16966 and AVF2107g trials.

## Materials and methods

Patient populations, treatment, and outcomes have been described in the AVF2107g and NO16966 studies.^10,11^ Briefly, AVF2107g was a randomised, placebo-controlled trial of bevacizumab with irinotecan, bolus fluorouracil, and leucovorin in patients with previously untreated mCRC.^10^ NO16966 was a randomised, noninferiority comparison of 5-fluorouracil, folinic acid, and oxaliplatin (FOLFOX4) vs capecitabine plus oxaliplatin (XELOX), which was subsequently amended to a 2 × 2 factorial design with further randomisation to bevacizumab or placebo.^11^

The aforementioned analysis by Loupakis et al.^8^ which included data from AVF2107g and NO16966, evaluated the association between tumour location and survival in mCRC. Thus, all evaluable patients from AVF2107g and both part 1 (FOLOX4 vs XELOX) and part 2 (2 × 2 factorial plus bevacizumab) of NO16966 were included. However, since our objective was to compare the effect of bevacizumab + CT vs CT in right- and left-sided tumours, our analysis focuses on patients who were concurrently randomised to bevacizumab + CT. Patients who received CT only (FOLFOX4 or XELOX) from part 1 of NO16966 are excluded here.

Sidedness of patients from NO16966 (NCT00069095) and AVF2107g (NCT00109070) was identified by Clinical Study Report information, including surgery procedural reports, updated from Loupakis et al.^8^ Cancers proximal (i.e. occurring in the caecum, ascending colon, or transverse colon) or distal (i.e., occurring in the descending colon, sigmoid colon, or rectum) to the splenic flexure were defined as right- or left-sided, respectively. Tumours occurring precisely at the splenic flexure were assigned to the left-sided group. Patients with synchronous right- and left-sided tumours were excluded. Median progression-free survival (PFS) and OS, as well as corresponding 95% CIs, were estimated using Kaplan-Meier methods. Interaction tests were performed to determine whether treatment outcomes with bevacizumab + CT vs CT were different for patients with right- vs left-sided mCRC tumours. Unadjusted Cox proportional hazards models with right/left terms were used to compare survival outcomes between treatment groups and were correlated with efficacy endpoints; corresponding HRs and 95% CIs were estimated. In an overall treatment comparison for all patients with right- or left-sided tumours, tumour location was included as a covariate in the Cox model and interaction of treatment and tumour location was assessed. Overall response rate (ORR) was summarised as categorical data and compared between treatment groups by odds ratio (OR).

## Results

**Fig. 1:**
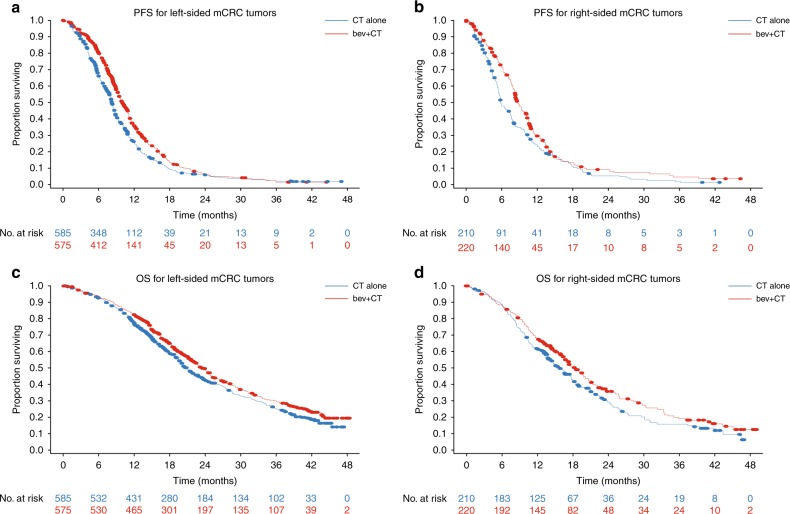
Kaplan-Meier PFS and OS curves by tumour side for pooled population. **a** PFS for left-sided. **b** PFS for right-sided tumours. **c** OS for left-sided. **d** OS for right-sided. Bev bevacizumab, CT chemotherapy, OS overall survival, PFS progression-free survival

Sidedness was determined in 1590 patients (27% right and 73% left) of 2214 patients total (Table 1). PFS was superior in patients treated with bevacizumb + CT vs CT in both right- (median PFS, 8.7 vs 5.8 months; HR = 0.75; 95% CI 0.61, 0.93; *p* = 0.008) and left-sided (median PFS, 10.0 vs 8.2 months; HR = 0.76; 95% CI 0.67, 0.86; *p* < 0.001) mCRC in this pooled analysis of NO16966 and AVF2107g (Fig. 1a, b). OS was also numerically improved with bevacizumab + CT vs CT in right-sided mCRC (median OS, 18.3 vs 15.6 months; HR = 0.82; 95% CI 0.65, 1.03; *p* = 0.085) and statistically significantly improved in left-sided mCRC (median OS, 23.5 vs 20.8 months; HR = 0.85; 95% CI 0.74, 0.98; *p* = 0.028) (Fig. 1c, d). The magnitude of PFS and OS benefit did not differ by tumour location.

**Table 1:** Survival outcomes

	PFS		OS	
	CT	Bev + CT	CT	Bev + CT
*Right side*				
NO16966				
*N*	107	117	107	117
Median (95% CI)	7.6 (5.9, 9.9)	8.6 (7.6, 10.2)	17.7 (14.7, 21.0)	20.1 (17.5, 23.5)
AVF2107				
*N*	103	103	103	103
Median (95% CI)	5.4 (4.4, 5.8)	8.7 (8.1, 10.6)	13.6 (10.6, 16.7)	15.9 (12.7, 19.6)
Pooled				
*N*	210	220	210	220
Median (95% CI)	5.8 (5.4, 7.4)	8.7 (8.2, 10.1)	15.6 (13.6, 17.9)	18.3 (16.0, 20.6)
HR (95% CI) *p* value	0.75 (0.61, 0.93) 0.008		0.82 (0.65, 1.03) 0.085	
*Left side*				
NO16966				
*N*	386	380	386	380
Median (95% CI)	8.5 (8.0, 9.0)	9.6 (9.2, 10.2)	22.4 (20.5, 24.6)	23.3 (21.2, 24.8)
AVF2107				
*N*	199	195	199	195
Median (95% CI)	7.8 (5.7, 8.2)	11.0 (10.2, 13.0)	16.4 (15.3, 19.6)	24.2 (19.9, NR)
Pooled				
*N*	585	575	585	575
Median (95% CI)	8.2 (7.9, 8.5)	10.0 (9.4, 10.8)	20.8 (19.6, 22.4)	23.5 (21.6, 24.8)
HR (95% CI) *p* value	0.76 (0.67, 0.86) <0.001		0.85 (0.74, 0.98) 0.028	

*Bev* bevacizumab, *CI* confidence interval, *CT* chemotherapy, *HR* hazard ratio, *PFS* progression-free survival, *OS* overall survival

**Fig. 2:**
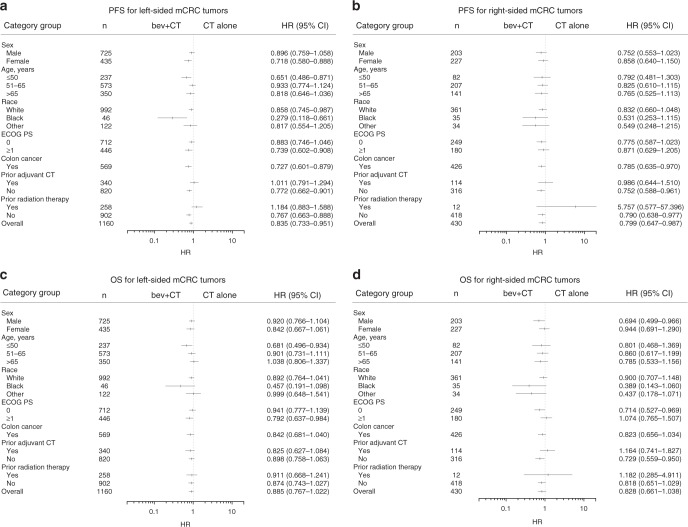
Forest plots for PFS and OS by tumour side for pooled population. **a** PFS for left-sided. **b** PFS for right-sided. **c** OS for left-sided. (**d**) OS for right-sided. Bev, bevacizumab; CT, chemotherapy; OS, overall survival; PFS, progression-free survival

To determine survival outcomes by baseline characteristics, patients were stratified by sex, age, race, Eastern Cooperative Oncology Group performance status, presence of colon cancer, prior adjuvant CT, and prior radiation therapy. Compared with CT, bevacizumab + CT was associated with greater PFS and OS benefits for both right- and left-sided mCRC for most of these categories (Fig. 2).

In an overall treatment comparison for all patients with right- or left-sided tumours, tumour location was included as a covariate in the Cox model and interaction of treatment and tumour location was non-significant (PFS, *p* = 0.898; OS, *p* = 0.697). Cox model analysis also showed a non-significant (PFS, *p* = 0.980; OS, *p* = 0.879) interaction of bevacizumab use and availability of sidedness information (right/left vs. not classified). Interaction tests for PFS (*p* = 0.904) and OS (*p* = 0.689) indicated that treatment effect did not differ between patients based on tumour location. ORRs were similar with bevacizumab + CT vs CT in both right-sided (40.9% vs 42.4%; OR, 0.94; 95% CI 0.64–1.38; *p* = 0.770) and left-sided (54.4% vs 52.0%; OR, 1.10; 95% CI 0.88–1.39; *p* = 0.410) mCRC.

## Discussion

Right-sided tumours have been widely associated with worse prognosis compared with left-sided tumours in mCRC.^8,12–14^ Meta-analysis showed that left-sided CRC was associated with a 20% reduction in the risk of death vs right-sided CRC, independent of disease stage, ethnicity, and study type.^12^ In our retrospective subgroup analysis of two randomised phase III mCRC studies, bevacizumab + CT improved survival vs CT alone, and the effect of bevacizumab was independent of tumour location. Similar improvements in PFS and OS were observed when patients were stratified according to baseline characteristics, such as age or race. In addition, OS was numerically improved with bevacizumab + CT vs CT in right-sided mCRC (median OS, 18.3 vs 15.6 months; *p* = 0.085) and significantly improved in left-sided mCRC (median OS, 23.5 vs 20.8 months; *p* = 0.028). However, it is likely that the smaller magnitude of the right-sided mCRC patient subset contributed to the non-significant *p* value for OS; therefore, these data suggest survival benefit overall and for both right- and left-sided tumours.

This is the first analysis of two large phase III mCRC studies comparing bevacizumab + CT vs CT alone by tumour location. As such, it represents a more comprehensive analysis of the effect of bevacizumab by tumour location than previous reports, which have been mixed.^15–17^ For instance, a retrospective analysis of mCRC patients treated with XELOX with or without bevacizumab suggested that bevacizumab may primarily benefit patients with left-sided primary tumours.^16^ However, in another study, PFS was superior with bevacizumab + CT vs CT regardless of tumour site (HR = 0.46).^15^ Our results build upon findings from the study by Loupakis et al.^8^ which showed that primary tumour location has a strong prognostic effect on patients with mCRC irrespective of bevacizumab exposure.

This study was a retrospective subgroup analysis, and was therefore limited to the patient populations investigated in the AVF2017g and NO16966 trials.^10,11^ The tumour location of all patients was not identifiable; however, interaction tests showed no evidence of a relationship between the ability to identify the side and outcomes (PFS interaction, *p* = 0.980; OS interaction, *p* = 0.879). Although both AVF2107g and NO16966 investigated bevacizumab, the CT backbones were different, which may have influenced the pooled results in our study. Another limitation is that data on RAS mutation status could not be included in our analysis. Since these data were not collected for NO16966, samples are not currently available for testing; for AVF2107g, sidedness information was only available for 180 out of 230 patients for whom KRAS data were collected,^18^ making the sample size too small for a valid analysis of the impact of treatment and RAS status. However, KRAS status has previously been shown not to be predictive for bevacizumab efficacy.^19^ Recent evidence points to the value of defining the sublocation of mCRC tumours, within the right- or left-sided classification method. In a study of 1876 CRC patients, the prevalence of mutations in *BRAFV600*, *TP53*, *PIK3CA*, and other genes differed by sublocation within right- and left-sided tumours.^20^ The authors concluded that the sigmoid-rectal region of the left side appears unique, and the transverse colon is distinct from other right-sided locations. In our study, the lack of data on precise tumour location could be seen as a limitation. However, given that bevacizumab efficacy is maintained in both right- and left-sided tumours, further analyses by tumour sublocation would likely not influence the data presented here.

This retrospective exploratory subgroup analysis of two pivotal studies indicates the effect of bevacizumab is independent of tumour location in a non-biomarker selected mCRC population.

## Data Availability

Qualified researchers may request access to individual patient level data through the clinical study data request platform (www.clinicalstudydatarequest.com). Further details on Roche’s criteria for eligible studies are available here (https://clinicalstudydatarequest.com/Study-Sponsors/Study-Sponsors-Roche.aspx). For further details on Roche’s Global Policy on the Sharing of Clinical Information and how to request access to related clinical study documents, see here (https://www.roche.com/research_and_development/who_we_are_how_we_work/clinical_trials/our_commitment_to_data_sharing.htm).

## References

[1] Arnold D (2017). Prognostic and predictive value of primary tumour side in patients with RAS wild-type metastatic colorectal cancer treated with chemotherapy and EGFR directed antibodies in six randomised trials. Ann. Oncol..

[2] Meza R, Jeon J, Renehan AG, Luebeck G (2010). Colorectal cancer incidence trends in the United States and United Kingdom: evidence of right- to left-sided biological gradients with implications for screening. Cancer Res..

[3] Missiaglia E (2014). Distal and proximal colon cancers differ in terms of molecular, pathological, and clinical features. Ann. Oncol..

[4] Benedix F (2010). Comparison of 17,641 patients with right- and left-sided colon cancer: differences in epidemiology, perioperative course, histology, and survival. Dis. Colon Rectum.

[5] Meguid RA, Slidell MB, Wolfgang CL, Chang DC, Ahuja N (2008). Is there a difference in survival between right- versus left-sided colon cancers?. Ann. Surg. Oncol..

[6] Dejea CM (2014). Microbiota organization is a distinct feature of proximal colorectal cancers. Proc. Natl Acad. Sci. USA.

[7] Mik M, Berut M, Dziki L, Trzcinski R, Dziki A (2017). Right- and left-sided colon cancer—clinical and pathological differences of the disease entity in one organ. Arch. Med. Sci..

[8] Loupakis F (2015). Primary tumor location as a prognostic factor in metastatic colorectal cancer. J. Natl Cancer Inst..

[9] Tejpar S (2017). Prognostic and predictive relevance of primary tumor location in patients with RAS wild-type metastatic colorectal cancer: retrospective analyses of the CRYSTAL and FIRE-3 trials. JAMA Oncol..

[10] Hurwitz H (2004). Bevacizumab plus irinotecan, fluorouracil, and leucovorin for metastatic colorectal cancer. N. Engl. J. Med..

[11] Saltz LB (2008). Bevacizumab in combination with oxaliplatin-based chemotherapy as first-line therapy in metastatic colorectal cancer; a randomized phase III study. J. Clin. Oncol..

[12] Petrelli F (2017). Prognostic survival associated with left-sided vs right-sided colon cancer: a systematic review and meta-analysis. JAMA Oncol..

[13] Modest DP (2014). Outcome of patients with metastatic colorectal cancer depends on the primary tumor site (midgut vs. hindgut): analysis of the FIRE1-trial (FuFIRI or mIROX as first-line treatment). Anticancer Drugs.

[14] Yahagi M, Okabayashi K, Hasegawa H, Tsuruta M, Kitagawa Y (2016). The worse prognosis of right-sided compared with left-sided colon cancers: a systematic review and meta-analysis. J. Gastrointest. Surg..

[15] Wong HL (2016). Impact of primary tumor site on bevacizumab efficacy in metastatic colorectal cancer. Clin. Colorectal Cancer.

[16] Boisen MK (2013). Primary tumor location and bevacizumab effectiveness in patients with metastatic colorectal cancer. Ann. Oncol..

[17] Holch JW, In Ricard, Stintzing S, Modest DP, Heinemann V (2017). The relevance of primary tumour location in patients with metastatic colorectal cancer: a meta-analysis of first-line clinical trials. Eur. J. Cancer.

[18] Hurwitz HI, Yi J, Ince W, Novotny WF, Rosen O (2009). The clinical benefit of bevacizumb in metastatic colorectal cancer is independent of K-ras mutation status: analysis of a phase III study of bevacizumab with chemotherapy in previously untreated metastatic colorectal cancer. Oncologist.

[19] Hurwitz HI (2013). Efficacy and safety of bevacizumab in metastatic colorectal cancer: pooled analysis from seven randomized controlled trials. Oncologist.

[20] Loree JM (2018). Classifying colorectal cancer by tumor location rather than sidedness highlights a continuum in mutation profiles and Consensus Molecular Subtypes. Clin. Cancer Res..

